# Heat stress assessment in chickens via key head region temperature measurement using semantic segmentation and cross-modal RGB-IR collaboration

**DOI:** 10.1016/j.psj.2025.106151

**Published:** 2025-11-24

**Authors:** Yilei Hu, Jinming Pan, Lin Yu, Di Cui

**Affiliations:** a College of Biosystems Engineering and Food Science, Zhejiang University, Hangzhou 310058, PR China; b Hefei Shenmu Information Technology Co., Ltd., Hefei 230601, PR China

**Keywords:** Chicken, Cross-modal collaboration, Heat stress, Semantic segmentation, Temperature measurement

## Abstract

Surface body temperature is an important indicator for assessing heat stress in chickens, and infrared (IR) thermography is an effective method for obtaining it. However, current methods for selecting temperature measurement regions of interest (ROIs) in IR images often struggle to accurately and comprehensively reflect the actual size and shape of the measured areas, inevitably introducing noise from background or irrelevant areas during the temperature acquisition process. This study proposes a cross-modal RGB-IR collaborative segmentation framework. It utilizes the Mask2Former model to remove the background from RGB images and the key chicken head region semantic segmentation (CHSFormer) model to extract the ROIs of the comb, eye, beak, and wattle. The ROIs are then mapped to the corresponding regions in the IR images using the cross-modal coordinate mapping algorithm (CCMA), which enables accurate temperature measurement of the key chicken head regions. RGB-IR images of chickens were used in experiments to evaluate the performance of the framework. The results showed that the Mask2Former for background segmentation achieved mean intersection over union (MIoU) and mean pixel accuracy (MPA) of 99.05 % and 99.47 %, respectively. The CHSFormer for key chicken head region segmentation achieved MIoU and MPA of 93.21 % and 96.49 %, respectively. The CCMA accurately mapped the ROIs of key chicken head regions from the RGB images to the corresponding regions in the IR images. The temperature measurement results indicated that the temperatures of the comb and wattle significantly increased under high-temperature environments, which effectively reflected the heat stress in chickens.

## Introduction

China is the world’s largest producer and consumer of poultry meat, with poultry consumption accounting for approximately 25 % of total meat intake. As an essential component of poultry farming, broiler chicken production plays a critical role in ensuring poultry meat supply, promoting agricultural economic development, and increasing farmers’ incomes (China Animal Agriculture Association, 2022). The prevalent practice in China employs a “companies + farmers” contract farming model, whereby farmers typically raise broiler chickens provided by companies in semi-open broiler houses using the mode of floor rearing. This approach is characterized by high rearing density and low production costs, yet it generally lacks efficient and reasonable environmental control measures. Consequently, in hot summer conditions in the southeastern coastal regions of China, where temperatures can reach around 39 °C, the temperature distribution within the broiler houses is often uneven, with localized temperatures exceeding the optimal range for chicken growth, thereby inducing heat stress in the chickens (Slawinska et al., 2020). Heat stress significantly reduces chickens’ feed intake, body weight gain, growth rate, meat quality, and overall health and welfare (Apalowo et al., 2024; Shakeri et al., 2019; Wasti et al., 2020). With the ongoing upward trend in global average temperatures and an increased frequency of extreme heat events due to climate change (IPCC, 2023), the heat stress issue in broiler chicken production has become increasingly severe. Currently, the on-farm assessment of heat stress in chickens largely depends on manual observation, which is subjective, labor-intensive, and requires a high level of expertise among farm workers. While laboratory tests, such as measuring plasma corticosterone levels or the concentrations of serum heat shock proteins (HSPs), offer more objective insights, they are not always readily available on farms and may take too long to yield results (Hu et al., 2021; Mahmoud et al., 2004). This approach struggles to meet the demands of modern, sustainable poultry farming (He et al., 2022). Therefore, there is an urgent need to develop an objective, accurate, and reliable heat stress monitoring technology for chickens to assist farmers in implementing precise environmental regulation and targeted intervention strategies, which safeguards the production performance of chickens and economic returns for farmers.

Upon exposure to the stressors, the surface temperature of poultry changes significantly, making it an important indicator for heat stress assessment (Giloh et al., 2012; Tabh et al., 2021). Infrared (IR) thermography, as an emerging non-destructive detection technology, offers an efficient, non-invasive, and non-stressful means to obtain surface temperatures of animals (Cai et al., 2023). IR thermography works by detecting the infrared radiation naturally emitted from an animal's body surface, which varies with its temperature. IR cameras capture this radiation and convert it into thermal images, mapping pixel intensities to temperature values, enabling accurate assessment without causing stress (Mota-Rojas et al., 2021). Researchers have adopted an improved U-Net (a convolutional neural network architecture for image segmentation) to automatically segment the eye and ear regions in the IR images of cows to measure their temperatures (Shu et al., 2024). This approach yields small mean differences between the acquired eye and ear temperatures and the actual values. Similarly, the forehead and ear base temperatures of pigs have a high correlation with rectal temperatures. By designating these as regions of interest (ROIs) for IR thermography, the object detection algorithm can automatically extract forehead and ear base temperatures from IR images, achieving mean absolute errors (MAE) of 0.098 ℃ and 0.083 ℃, respectively, compared to actual temperatures (Xie et al., 2023). In addition, the ocular temperature of pigs is closely correlated with core body temperature, and IR ocular temperature can serve as a non-invasive indicator to replace rectal temperature for monitoring heat stress in pigs (Ricci et al., 2019).

Compared to the body surface temperature measurement of larger animals such as cattle and pigs, the measurement in poultry is complicated by feather coverage. The directly measured surface temperature may deviate significantly from the true skin surface temperature. In severely feather-damaged areas of chickens, the mean surface temperature is over 10 °C higher than that of the feather-covered back region (Yang et al., 2022). However, the chicken head and feet, which are naturally featherless, provide regions where temperature changes can serve as direct indicators of physiological status and health (Sadeghi et al., 2023). The ROIs of chicken head and feet in IR images can be selected manually or automatically through object detection algorithms, thus acquiring their temperatures from the temperature matrices (Uemura et al., 2023). The surface temperature of the chicken head can be extracted from both top and side IR views. While top views reveal the overall dorsal temperature of the head, side views provide more detailed information, which allows for further extraction of the eye, comb, and wattle temperatures. In contrast, the temperature of the chicken feet is typically only obtainable from the side view (Xiong et al., 2019; Xu et al., 2021). Comparative analysis of ROI temperatures under various environmental stresses and physiological states can thus rapidly assess whether chickens are healthy, suffering from heat or cold stress, or potentially diseased (Bloch et al., 2020).

However, IR images reflect only the thermal radiation from the animal surface and often lack clear texture and local contour details. Current ROI selection methods in IR images frequently struggle to accurately and comprehensively reflect the actual size and shape of the measured areas, inevitably introducing noise from background or irrelevant areas during the temperature acquisition process. For example, box-based ROI selection in the IR images during extraction of head and comb temperatures may include background areas, which is inadequate for accurately reflecting the true target temperature (Edgar et al., 2013; Wang et al., 2025). Similarly, although some studies attempt to align IR images with RGB images and use object detection to locate pig eye regions in the RGB images for temperature extraction (Zhang et al., 2024), rectangular ROIs still fail to precisely conform to the eye region and inevitably introduce background noise. This approach is thus more suited for measuring “periorbital” temperature rather than the “eye” itself. In other cases, manual ROI selection has been used for the eye, comb, and wattle (Solis et al., 2024), where the “eye” ROI actually measures periorbital temperature, and the comb and wattle ROIs reflect more accurately the true temperature because they are located within the interior of these regions. Overall, box-based ROI selection using object detection is prone to introducing background noise, failing to meet accuracy requirements, while manual ROI selection is laborious and inefficient, failing to satisfy the demand for efficiency.

To address the above issues, the general objective of this study is to develop an RGB-IR cross-modal collaborative segmentation framework, which maps the ROIs of key chicken head regions segmented from RGB images to the corresponding regions in IR images to obtain temperatures. The framework fully utilizes the rich texture and contour details in RGB images, obtains more accurate and complete target masks based on a semantic segmentation model, and uses a cross-modal coordinate mapping algorithm (CCMA) to generate the corresponding target masks in IR images for temperature measurement, which ultimately enables the assessment of heat stress in chickens. The specific objectives are: 1) to collect RGB-IR images of chickens with three different rearing densities and four different ages under normal and high temperature environments; 2) to propose an RGB-IR cross-modal collaborative segmentation framework; 3) to evaluate the performance of each component of the RGB-IR cross-modal collaborative segmentation framework as well as the overall performance of the framework; and 4) to extract the temperature of key chicken head regions based on the framework and assess the heat stress in chickens. This framework serves as an effective tool for heat stress monitoring in poultry, which helps facilitate timely interventions to prevent heat stress, optimize environmental controls in poultry houses, and enhance animal welfare and production efficiency.

## Materials and methods

### Experimental setup and data acquisition

**Fig. 1:**
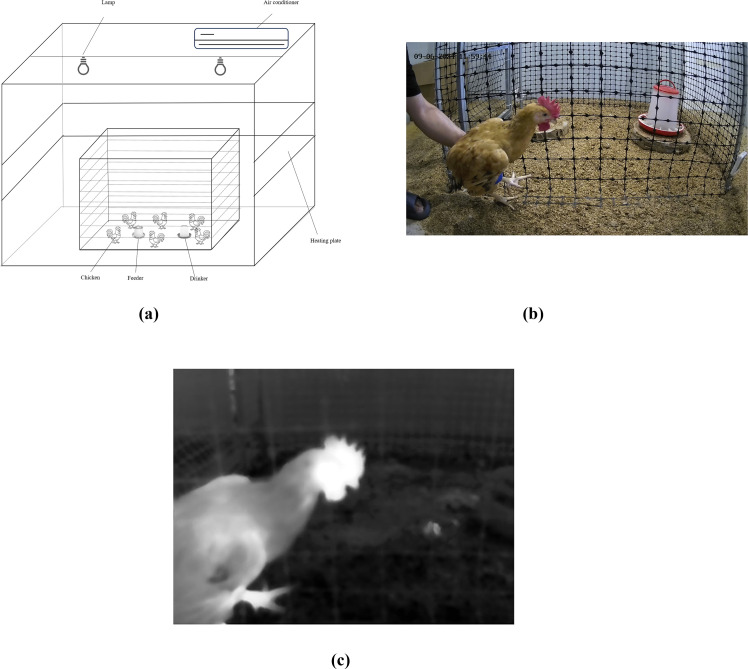
Experimental environment and acquired RGB-IR image pairs. (a) Experimental environment. (b) Acquired RGB image. (c) Acquired IR image.

The study involved 320 male chickens of the Youhuang 5B breed. All chickens were purchased at 20 days of age from Jiangsu Lihua Co., Ltd., and were housed in a room (room 0) equipped with comprehensive environmental control, feeding, and lighting systems, at a rearing density of 8 birds/m². All procedures of this experiment were performed under the guidance of the Care and Use of Animals of Zhejiang University (Hangzhou, China). The protocol was approved by the Committee on the Ethics of Animal Experiments of Zhejiang University. As shown in Fig. 1(a), the experiments were conducted in six separate but identical rooms (Rooms 1-6), each measuring 3.2 m in length, 3.0 m in width, and 2.6 m in height. Each room was equipped with a heating plate, air conditioner, and temperature-humidity sensors, and the floor was covered with a 5-cm-thick bedding layer. The light was on from 6:00 to 22:00 and automatically controlled by a timer, with an intensity of 25 lux. The chickens' activity range was restricted to a 1.0 *m* × 1.0 m area in the center of the room by a fence, where feeders and drinkers were provided. When the chickens in room 0 reached 28, 35, 42, and 49 days of age, 4 chickens were randomly selected and transferred to each of Rooms 1 and 2, where they were reared at a density of 4 birds/m² for three days; 8 chickens were transferred to each of Rooms 3 and 4 at a density of 8 birds/m² for three days; and 12 chickens were transferred to each of Rooms 5 and 6 at a density of 12 birds/m² for three days. This arrangement ensured that the chickens in each room adapted to the new environment. The experiments were conducted when the chickens in Rooms 1–6 reached 31, 38, 45, and 52 days of age. Each day, from 12:00 to 16:30, the environmental temperature in Rooms 1, 3, and 5 was set to 35 ± 0.5 °C to induce heat stress responses in the chickens (Valle et al., 2021), while the temperature in Rooms 2, 4, and 6 was maintained at 23 ± 0.5 °C. All other conditions in the rooms were kept consistent, as shown in Table 1. Additionally, the mean body weights of chickens across different age weeks during the experiment are presented in Table S1.

**Table 1:** Temperature, rearing density, and age settings for rooms 1-6.

Groups	Room	Temperature (℃)	Density (birds/m²)	Weeks of age (w)
Experimental Group	Room 1	35 ± 0.5	4	5, 6, 7, 8
Control Group	Room 2	23 ± 0.5	4	
Experimental Group	Room 3	35 ± 0.5	8	
Control Group	Room 4	23 ± 0.5	8	
Experimental Group	Room 5	35 ± 0.5	12	
Control Group	Room 6	23 ± 0.5	12	

During the experiment, RGB-IR images of the chickens and the corresponding temperature matrices were collected at 12:00 and 16:30 using a dual-channel camera (DS-2TD2636B-10/P (B), Hikvision, Hangzhou, China), as shown in Fig. 1, Fig. 1. The resolution of the RGB channel of the camera was 2688 × 1520, and the IR channel was 384 × 288, with an accuracy of ±0.5 °C and an emissivity setting of 0.96. The camera was fixed at a height of 500 mm above the ground and positioned at a 20° pitch angle downward relative to the horizontal direction. During image acquisition, the distance between the chicken and the RGB channel of the camera was fixed at 600 mm. This imaging setup was consistently applied in both the experimental and control groups to ensure fair comparisons. Specifically, one experimenter held the chicken by its legs on one side to immobilize it, while another experimenter used a ruler (accuracy: 1 mm) to measure and position the chicken’s head at a distance of 600 mm from the RGB channel. After positioning, the experimenter with the ruler left the camera’s field of view to reduce background complexity, and multiple sets of RGB-IR image pairs were captured consecutively for each chicken. In this way, RGB-IR images were obtained for chickens of 3 different rearing densities and 4 different ages in both the experimental and control groups. Only images with clear focus and standard side-view head poses were selected for further analysis.

In this study, two datasets were constructed, which included a background segmentation dataset and a key chicken head region semantic segmentation dataset. The background segmentation dataset consisted of 200 RGB images of chickens, sampled from all 6 experimental rooms across the 4 different ages. Since all rooms had identical internal setups and the RGB images collected in the laboratory had a structured and uniform background, background segmentation was a relatively simple task. Thus, these 200 images were deemed sufficient for this task and helped mitigate the risk of model overfitting. The key chicken head region semantic segmentation dataset consisted of 498 RGB images, each containing 5 semantic regions: background, comb, eye, beak, and wattle. These images were sampled from all age, density, and temperature combinations to ensure representativeness and enhance the model's generalizability across various conditions. The background segmentation dataset and the key chicken head region semantic segmentation dataset were used to train and test the Mask2Former (Cheng et al., 2021a) and key chicken head region semantic segmentation model (CHSFormer, Section *Background segmentation model and key chicken head region semantic segmentation model*), respectively. The reason for selecting Mask2Former as the baseline model was its advanced transformer-based architecture, which provided a strong foundation for integration and further improvement in our tasks. In addition, 298 pairs of RGB-IR images, obtained from chickens at 3 different rearing densities and 4 different ages, were used for the implementation and performance evaluation of the CCMA (Section *Cross-modal coordinate mapping algorithm*).

### Algorithm development environment and software

The algorithm development and testing platform used in this study is a computer equipped with an NVIDIA GeForce RTX 4090 (GPU) with 24GB of VRAM, a 14th Gen Intel(R) Core(TM) i9-14900 (CPU), and the Ubuntu 22.04 operating system. The project environment includes Python 3.10, OpenCV 4.8.1, Torch 2.0.0, Torchvision 0.15.1, and CUDA 11.7. Labeling of the background segmentation dataset and the key chicken head region semantic segmentation dataset was performed using LabelMe v5.5.0. The temperature matrices were extracted using the software provided with the Hikvision dual-channel camera.

### RGB-IR cross-modal collaborative segmentation framework

**Fig. 2:**
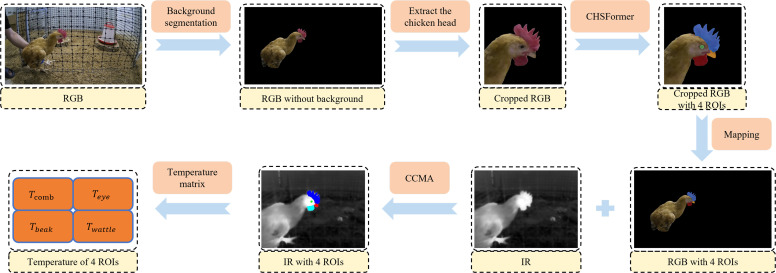
RGB-IR cross-modal collaborative segmentation framework.

***Overall framework*** The proposed RGB-IR cross-modal collaborative segmentation framework is shown in Fig. 2. For each collected RGB-IR image pair, the background segmentation model was first applied to the RGB image to segment the chicken from the background. The image was then cropped to retain only the complete head region of the chicken. The CHSFormer model was subsequently used to perform semantic segmentation of the key chicken head regions, which included the comb, eye, beak, and wattle. The masks of these four key regions were mapped back onto the background-free RGB image. Based on the four mask regions in the background-free RGB image, CCMA was used to map these regions to the corresponding areas in the IR image of the RGB-IR image pair. Finally, in combination with the temperature matrix, the temperatures of the comb, eye, beak, and wattle in the IR image were obtained.

***Cross-modal coordinate mapping algorithm*** The principle of CCMA in the RGB-IR cross-modal collaborative segmentation framework is described as follows. First, the contour point sets of the four key regions—the comb, eye, beak, and wattle—are extracted from the background-free RGB image. For each contour point in the mask region, distortion correction is performed based on the distortion parameters $D_{rgb}$ of the RGB channel to obtain the corrected pixel coordinates $\left(u_{rgb},v_{rgb}\right)$. These are then normalized using the intrinsic matrix ​$K_{rgb}$ of the RGB channel to obtain the undistorted normalized pixel coordinates $\left(x_{norm},y_{norm}\right)$, as shown in Eq. (1).

(1) $$\left(x_{norm},y_{norm}\right)=\left(\frac{u_{rgb}-c_{xrgb}}{f_{xrgb}},\frac{v_{rgb}-c_{yrgb}}{f_{yrgb}}\right)$$

where $u_{rgb}$ and $v_{rgb}$ are the pixel coordinates, $f_{xrgb}$ and $f_{yrgb}$ are the focal lengths, and $c_{xrgb}$ and $c_{yrgb}$ are the principal point coordinates of the RGB channel.

Next, the normalized coordinates $\left(x_{norm},y_{norm}\right)$ are converted to 3D points $P_{rgb}$ in the RGB camera coordinate system, as shown in Eq. (2).

(2) $$P_{rgb}=\left(x_{norm}\cdot depth,\;y_{norm}\cdot depth,\;depth\right)$$

where depth represents the distance from the RGB channel to the target plane.

Then, the 3D point $P_{rgb}$​ in the RGB channel coordinate system is mapped to the 3D point $P_{ir}$ in the IR channel coordinate system using the extrinsic matrix ​$T_{rgb\_T\_ir}$ of the dual-channel camera, as shown in Eq. (3).

(3) $$P_{ir}=T_{rgb\_T\_ir}\cdot P_{rgb}$$

where $T_{rgb\_T\_ir}$ is the extrinsic matrix containing rotation and translation parameters for the spatial transformation from the RGB channel coordinate system to the IR channel coordinate system.

Subsequently, based on the intrinsic matrix $K_{ir}$ and distortion coefficients $D_{ir}$ of the IR channel, the 3D point $P_{ir}$ in the IR channel coordinate system is projected onto the IR image coordinate system to obtain the pixel coordinates $\left(u_{ir},v_{ir}\right)$, as shown in Eqs. (4 and 5).

(4) $$u_{ir}=f_{xir}\cdot \frac{X}{Z}+c_{xir}+\Delta_{distortion}$$

(5) $$v_{ir}=f_{yir}\cdot \frac{Y}{Z}+c_{yir}+\Delta_{distortion}$$

where *X, Y*, and *Z* are the coordinates of $P_{ir}$, $f_{xir}$ and $f_{yir}$ are the focal lengths, $c_{xir}$ and $c_{yir}$ are the principal points of the IR channel, and $\Delta_{distortion}$ is the distortion correction term.

Finally, the contour point sets of the comb, eye, beak, and wattle in the IR image are closed and filled to obtain the corresponding mask regions in the IR image.

The intrinsic and extrinsic parameters of the dual-channel camera used in CCMA, which included $D_{rgb}$, ​$K_{rgb}$, $D_{ir}$, $K_{ir}$, and $T_{rgb\_T\_ir}$​, were all obtained through Zhang’s checkerboard calibration method (Z., 2000). To successfully acquire IR images of the checkerboard, a black-and-white checkerboard pattern was coated with a layer of black chromium film on optical glass. The black chromium film (black squares) and the optical glass (white squares) together formed a 15 × 16 checkerboard array, with each square measuring 25 mm × 25 mm. A heating pad was placed under the checkerboard to ensure that the checkerboard corners were clearly visible in both the RGB and IR images (Zhang et al., 2023).

**Fig. 3:**
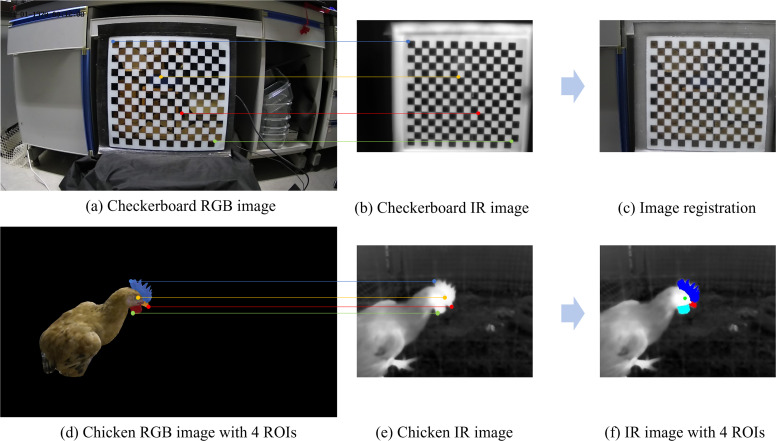
Example of the registration and ROI mapping for the cross-modal coordinate mapping algorithm CCMA. (a) Checkerboard RGB image. (b) Checkerboard IR image. (c) Image registration. (d) Chicken RGB image with 4 ROIs. (e) Chicken IR image. (f) IR image with 4 ROIs.

Fig. 3 illustrates the checkerboard image registration and chicken image ROI mapping using CCMA. Fig. 3(a)-(b) show the RGB and IR images of the checkerboard, respectively. Fig. 3(c) presents the registration result, where all the checkerboard corners are precisely matched in a one-to-one correspondence. Fig. 3(d)-(e) display the RGB and IR images of the chicken, respectively. Fig. 3(f) shows the mapped ROIs of the chicken head, which accurately reflect the contours and boundaries of the comb, eye, beak, and wattle.

However, the distance (depth) from the RGB channel to the target plane affects the mapping accuracy of CCMA. To systematically evaluate the impact of depth error on the accuracy of CCMA, this study used 600 mm as the baseline and traversed the depth interval [580, 620] mm in 1 mm increments. For all RGB-IR image pairs, CCMA was applied to obtain the mask regions of the comb, eye, beak, and wattle in the IR images at different depths. The mask regions at each depth were then quantitatively compared with those at the baseline depth. Specifically, for each RGB-IR image pair, the IoU and centroid offset of the four key region masks in the IR image at depth *d* (*d*∈[580,620]) were calculated relative to those at the baseline depth. The average IoU and average centroid offset for all test image pairs were then computed to reflect the impact of depth error on CCMA.

**Fig. 4:**
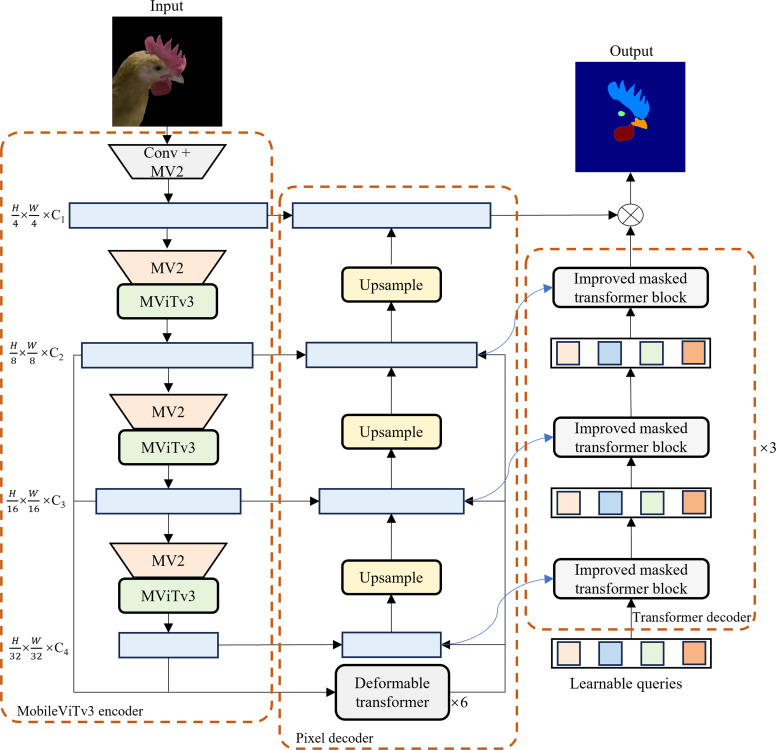
Architecture of the CHSFormer model for key chicken head region segmentation.

***Background segmentation model and key chicken head region semantic segmentation model*** The semantic segmentation models used in this study included the background segmentation model Mask2Former and the key chicken head region semantic segmentation model CHSFormer. The Mask2Former was used to remove the background from the chicken RGB images. To improve the segmentation accuracy of the comb, eye, beak, and wattle, we modified Mask2Former to develop the CHSFormer for key chicken head region semantic segmentation, the architecture of which is shown in Fig. 4. CHSFormer first utilizes a MobileViTv3 encoder to extract image features from the input chicken images, thereby obtaining multi-scale semantic feature maps. The pixel decoder then receives the last three feature maps from the encoder and performs multi-scale fusion and context modeling on these three feature maps through six groups of multi-scale deformable attention transformer encoders, which results in three feature-enhanced feature maps. An FPN-style upsampling and fusion strategy is adopted to progressively integrate these multi-scale features and increase spatial resolution, which ultimately generates high-resolution mask features that provide rich spatial detail for subsequent mask prediction. In the transformer decoder, the model introduces a set of learnable queries, which are continuously updated and optimized through three groups of improved masked transformer blocks, aggregating rich semantic and spatial information. Finally, the transformer decoder outputs a class prediction and a mask embedding for each query. Mask prediction is obtained by linearly combining the mask embedding of each query with the high-resolution mask features, which enables precise segmentation of different targets. This entire process fully leverages the global modeling capability of transformers and the spatial detail representation of pixel-level features, which endows CHSFormer with robust segmentation capability for the key chicken head regions. The specific improvement methods for CHSFormer are as follows.

We adopted the MobileViTv3 backbone as the encoder for the CHSFormer model. MobileViTv3 is a lightweight and efficient network with a vision transformer backbone. The core idea of MobileViTv3 is to combine the advantages of convolutional neural networks (CNNs) and vision transformers. By introducing transformer modules within local receptive fields, it enables efficient modeling of both local and global features (Wadekar and Chaurasia, 2022). The incorporation of the MobileViTv3 encoder can significantly reduce the number of parameters and computational complexity of CHSFormer, while maintaining the multi-scale representation capability of the feature extractor. This provides the decoder with high-quality feature maps containing fine-grained structural information and global contextual relationships, thereby ensuring the segmentation accuracy of the comb, eye, beak, and wattle.

**Fig. 5:**
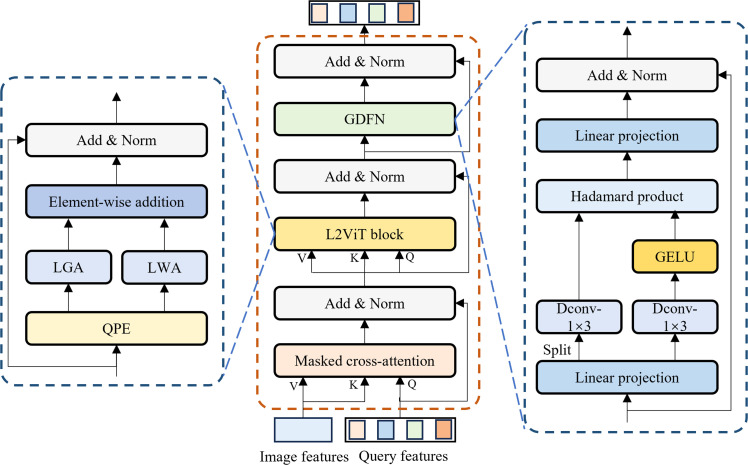
Structure of the improved masked transformer block in the transformer decoder of CHSFormer.

The structure of the improved masked transformer block is shown in Fig. 5. It consists of masked cross-attention, an L^2^ViT block, and a Gated-Dconv feed-forward network (GDFN). The masked cross-attention enables the queries to interact with feature maps of different resolutions from the pixel decoder, thereby integrating multi-scale spatial context. The L^2^ViT block is used to further model the global dependencies among the queries, while the GDFN enhances the feature representation capability of the queries. The L^2^ViT block is an efficient self-attention module that combines Local Window Attention (LWA) and Linear Global Attention (LGA) (Zheng, 2025). In the improved masked transformer block, the features from masked cross-attention, after normalization, are passed through Query Positional Embedding (QPE) and then fed into both the LWA and LGA modules, which capture local spatial information and global contextual information, respectively. The outputs of the two branches are summed, followed by residual connection and normalization to form the final output features. The advantage of this structure is that LWA can effectively model fine-grained information in spatially adjacent regions, while LGA expands the receptive field and enables long-range dependency modeling across regions. The combination of the two modules not only enhances the feature modeling capability but also alleviates the memory overhead of global self-attention. The integration of the L^2^ViT block can significantly improve computational efficiency while maintaining the ability to model global dependencies. The GDFN first increases the feature dimension through a linear transformation, then uses depthwise separable convolution to extract local contextual information. The output is then split along the channel dimension into two parts: one part is activated by GELU, while the other part is retained as is. The two parts are dynamically fused via a Hadamard product. The fused result is then projected back to the original channel dimension through a linear transformation, followed by residual connection and normalization (Zamir et al., 2021). Compared to the original FFN, GDFN can effectively enhance the model’s ability to analyze and discriminate local details in chicken images, which makes it suitable for the segmentation of key chicken head regions that require enhanced local fine-grained modeling.

The loss function of CHSFormer consists of a classification loss and a mask loss, where the classification loss adopts the cross-entropy loss function, and the mask loss adopts both Binary Cross-Entropy (BCE) Loss and Dice Loss. We replaced the BCE Loss in CHSFormer with Focal Loss (Lin et al., 2017), which is calculated as shown in Eq. (6). In the task of key chicken head region segmentation, the target regions occupy only a small proportion of the image and are easily affected by background noise. Focal Loss assigns higher loss weights to hard examples and suppresses the domination of easy examples in the total loss, thereby effectively alleviating the problem of small targets being dominated by large backgrounds. This enhances CHSFormer’s ability to segment small target regions and regions with blurry boundaries, and also improves the model’s convergence speed and stability during training.

(6) $$FL\left(p_{t}\right)=-\alpha {\left(1-p_{t}\right)}^{\gamma }\text{log}\left(p_{t}\right)$$

where $p_{t}$ represents the predicted probability of the true class, α is the class balance factor, and γ is the parameter that adjusts the loss contribution of easy and hard samples.

### Model training and testing

In this study, both the background segmentation dataset and the key chicken head region semantic segmentation dataset were randomly divided into training and test sets at a ratio of 4:1. These were used to train the background segmentation model Mask2Former and the key chicken head region semantic segmentation model CHSFormer, respectively, and to evaluate the performance of both models. During model training, the ADAMW optimizer was used to update the network weights and parameters (Loshchilov and Hutter, 2017). The hyperparameters that required fine-tuning included training epochs, batch size, input size, width multiplier of MobileViTv3, learning rate, and weight decay. Among these, the width multiplier of MobileViTv3 is a hyperparameter specific to CHSFormer, while the other hyperparameters were kept consistent for training both models, as shown in Table 2.

**Table 2:** Parameter settings for training the CHSFormer model.

Parameters	Values
Training epochs	50
Batch size	12
Input size	512
Width multiplier of MobileViTv3	1.25
Learning rate	0.0001
Weight decay	0.05

### Performance evaluation metrics

The model performance evaluation metrics used in this study included intersection over union (IoU), mean intersection over union (MIoU), and mean pixel accuracy (MPA). The calculation methods for MIoU and MPA are shown in Eqs. (7 and 8).

(7) $$MIoU=\frac{1}{k}\sum_{i=1}^{k}\frac{p_{ii}}{\sum_{j=1}^{k}p_{ij}+\sum_{j=1}^{k}p_{ji}-p_{ii}}$$

(8) $$MPA=\frac{1}{k}\sum_{i=1}^{k}\frac{p_{ii}}{\sum_{j=1}^{k}p_{ij}}$$

where *k* is the number of classes, $p_{ii}$ denotes the number of pixels that are truly of class *i* and are correctly predicted as class *i*, $p_{ij}$ denotes the number of pixels that are truly of class *i* but are misclassified as class *j*, and $p_{ji}$ denotes the number of pixels that are truly of class *j* but are misclassified as class *i*.

### Methods for measuring heat stress indicator and chicken head temperatures

To verify that the chickens indeed experienced heat stress during the experiment, the concentration of HSPs in chicken serum was quantified as a physiological stress indicator (Hartl et al., 2011; Hu et al., 2021). After the completion of each room experiment and the collection of RGB-IR images, four chickens were randomly selected for blood sampling from the brachial vein. The collected blood samples were left at room temperature for 30 minutes to allow clotting, after which the serum was separated, further purified by centrifugation, transferred to new centrifuge tubes, and labeled. The samples were then stored at −80 °C until all experiments were completed and subsequently sent for analysis. The serum concentrations of HSPs (MB-100045A) was quantified using the specific ELISA kits for chickens (Jiangsu Meibiao Biotechnology Co., Ltd, China). The differences in the concentrations of serum HSPs between the experimental and control groups at different ages were analyzed using t-tests to assess statistical significance. All statistical analyses were performed using Python 3.10.

Based on the RGB-IR cross-modal collaborative segmentation framework, the mean temperatures of the comb, eye, beak, and wattle at the end of the experiment (16:30) were calculated from all chickens under each combination of rearing density, age, and group, which provided biological replication and minimized individual variability.

## Results and discussion

### Performance analysis of RGB-IR cross-modal collaborative segmentation framework

***Results and analysis of the semantic segmentation model*** The performance of the background segmentation model Mask2Former and the key chicken head region semantic segmentation model CHSFormer was evaluated on the test set. Mask2Former achieved an MIoU of 99.05 % and an MPA of 99.47 % on the test set, which indicated its ability to perform high-precision segmentation of chickens against a structured background and to provide high-quality background-free chicken RGB images for the task of key chicken head region semantic segmentation. Table 3 presents the performance of the CHSFormer model before and after improvement. CHSFormer outperformed the original Mask2Former in the segmentation accuracy of all four key chicken head regions, which achieved particularly high IoU for the comb and wattle, at 95.19 % and 94.03 %, respectively. The IoU for the eye and beak improved significantly, by 1.5 % and 3.07 %, respectively. The MIoU and MPA of CHSFormer increased by 1.19 % and 0.26 %, respectively, while the number of parameters was reduced by 48.74 %. It was observed that the IoU for the eye and beak was slightly lower than that for the comb and wattle. This is mainly due to the presence of down feathers around the eyelids, which obscure the boundaries of the eyes. The intersection of the beak and the head is also covered by irregularly distributed feathers, and in some cases, the color of the beak is very similar to that of the feathers, which increases the difficulty of segmentation. Similarly, the IoU for the comb and wattle is difficult to further improve, as the roots of these regions are also obscured by feathers, which lead to semantic ambiguity in the model’s inference of their contour boundaries. However, the slight internal boundary ambiguity of the chicken head has almost no negative impact on the subsequent temperature measurement of key chicken head regions.

**Table 3:** Performance of CHSFormer before and after improvement.

Model	IoU^a^/%					MIoU^b^/%	MPA^c^/%	Params^d^/M
	Background	Comb	Eye	Beak	Wattle			
Mask2Former	99.45	94.60	88.77	83.98	93.32	92.02	96.23	47.44
CHSFormer	99.53	95.19	90.27	87.05	94.03	93.21	96.49	24.32

a IoU: Intersection over union.

b MIoU = Mean intersection over union.

c MPA = Mean pixel accuracy.

d Params/*M* = Parameters in millions.

To further validate the effectiveness of each key module in CHSFormer, a series of ablation experiments were conducted, and the results are shown in Table 4. In Experiment 2, introducing MobileViTv3 as the encoder reduced model parameters by 41.36 %, but MIoU slightly decreased due to its lower representational capacity compared to the original Swin Transformer encoder, despite its lightweight and efficient design. In Experiment 3, adding the L^2^ViT block improved both MIoU and MPA, as its local-global information interaction mechanism enhanced segmentation performance for fine-grained regions. In Experiment 4, incorporating GDFN into CHSFormer boosted segmentation performance and reduced parameters by 12.37 %, which demonstrated that GDFN effectively modeled local fine-grained features. In Experiment 5, replacing the BCE Loss in the mask loss with Focal Loss resulted in a 0.92 % increase in MIoU. When all the improved modules were used together (Experiment 6), the model achieved the highest MIoU and MPA, as well as the lowest parameter count. The synergistic effect of all modules not only enhanced the model’s representational capacity and segmentation accuracy but also significantly reduced the number of parameters, fully validating the effectiveness of the CHSFormer architecture.

**Table 4:** Contribution of different modules to the CHSFormer.

Experiment	MobileViTv3	L^2^ViT	GDFN^a^	Focal Loss	MIoU^b^/%	MPA^c^/%	Params^d^/M
1	×	×	×	×	92.02	96.23	47.44
2	√	×	×	×	91.95	96.27	27.82
3	×	√	×	×	92.74	96.40	49.81
4	×	×	√	×	92.41	96.61	41.57
5	×	×	×	√	92.94	95.66	47.44
6	√	√	√	√	93.21	96.49	24.32

a GDFN = Gated-Dconv feed-forward network.

b MIoU = Mean intersection over union.

c MPA = Mean pixel accuracy.

d Params/*M* = Parameters in millions.

To comprehensively evaluate the segmentation performance of CHSFormer, it was compared with other mainstream semantic segmentation models, as shown in Table 5. CHSFormer achieved the best results in both MIoU and MPA, reaching 93.21 % and 96.49 %, respectively. Its parameter count is only 24.32 M, which is significantly lower than most of the compared models. As a result, CHSFormer can significantly improve the segmentation accuracy of the comb, eye, beak, and wattle while greatly reducing the number of parameters. Compared to other models, including SegFormer (Xie et al., 2021), DeepLabv3 (Chen et al., 2017), PSPNet (Zhao et al., 2016), UPerNet (Xiao et al., 2018), FCN (Shelhamer et al., 2016), MaskFormer (Cheng et al., 2021b), EncNet (Zhang et al., 2018), CCNet (Huang et al., 2020), and Mask2Former (Cheng et al., 2021a), CHSFormer is more suitable for the segmentation of key chicken head regions and can provide reliable segmentation masks for temperature extraction of these regions.

**Table 5:** Performance comparison among different semantic segmentation models.

Model^a^	Encoder	MIoU^b^/%	MPA^c^/%	Params^d^/M
SegFormer	Mix Transformer-B0	91.70	95.90	3.72
Deeplabv3	Resnet50	91.33	95.38	70.46
PSPNet	Resnet50	91.63	95.72	51.33
UPerNet	Swin Transformer-Tiny	91.64	94.50	60.72
FCN	Resnet50	91.71	95.98	40.05
MaskFormer	Swin Transformer-Tiny	92.23	95.33	60.58
EncNet	Resnet50	91.43	95.15	38.24
CCNet	Resnet50	91.54	95.29	52.18
Mask2Former	Swin Transformer-Tiny	92.02	96.23	47.44
Ours	MobileViTv3	93.21	96.49	24.32

a Models are referenced as follows: SegFormer (Xie et al., 2021), DeepLabv3 (Chen et al., 2017), PSPNet (Zhao et al., 2016), UPerNet (Xiao et al., 2018), FCN (Shelhamer et al., 2016), MaskFormer (Cheng et al., 2021b), EncNet (Zhang et al., 2018), CCNet (Huang et al., 2020), and Mask2Former (Cheng et al., 2021a).

b MIoU = Mean intersection over union.

c MPA = Mean pixel accuracy.

d Params/*M* = Parameters in millions.

The superior performance of the CHSFormer model compared to other semantic segmentation models can be attributed to its tailored architecture optimized for the fine-grained segmentation of small and irregularly shaped chicken head regions. CHSFormer incorporates key enhancements, including the adoption of the lightweight MobileViTv3 backbone as the encoder, which efficiently captures both local details and global context. Additionally, the integration of modules like L^2^ViT for enhanced local-to-global feature interaction, GDFN for enhanced fine-grained modeling, and the Focal Loss for focusing on hard-to-classify samples and blurry boundaries. In contrast, traditional convolutional neural network models primarily rely on convolutional operations, which excel in local feature extraction but often struggle with capturing long-range dependencies, leading to lower accuracy in delineating intricate structures. While transformer-based models improve global context modeling through self-attention mechanisms, they may not be as specialized for domain-specific tasks like chicken head segmentation, where CHSFormer's customized improvements result in better boundary adherence and reduced misclassification, as evidenced by its highest MIoU and MPA.

**Fig. 6:**
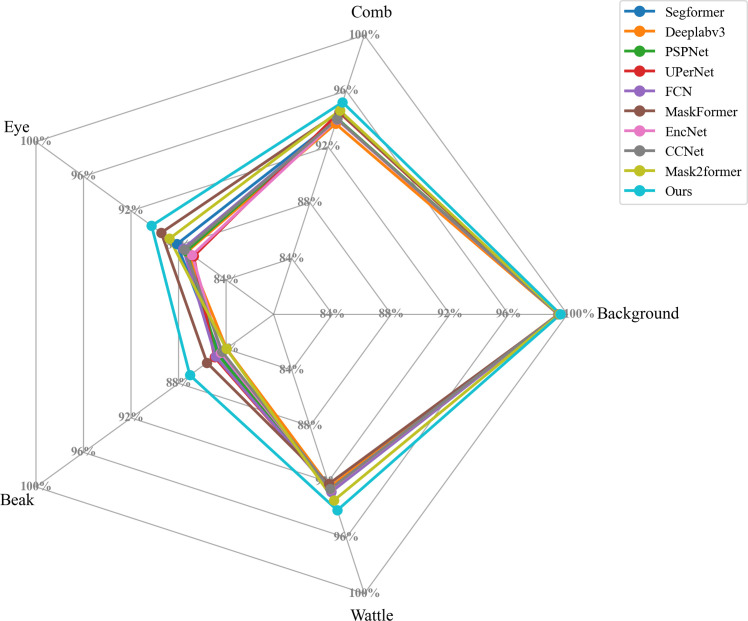
Comparison of IoU values for key chicken head regions among different semantic segmentation models.

Fig. 6 shows the radar charts of the IoU metrics for the five categories, which include background, comb, eye, beak, and wattle, across different models. Compared to other semantic segmentation models, CHSFormer achieved the best segmentation performance in all categories, with particularly higher IoU values for the eye and beak regions. This indicates that CHSFormer not only provides high overall segmentation accuracy but also demonstrates a clear advantage in segmenting each region of the chicken head.

**Fig. 7:**
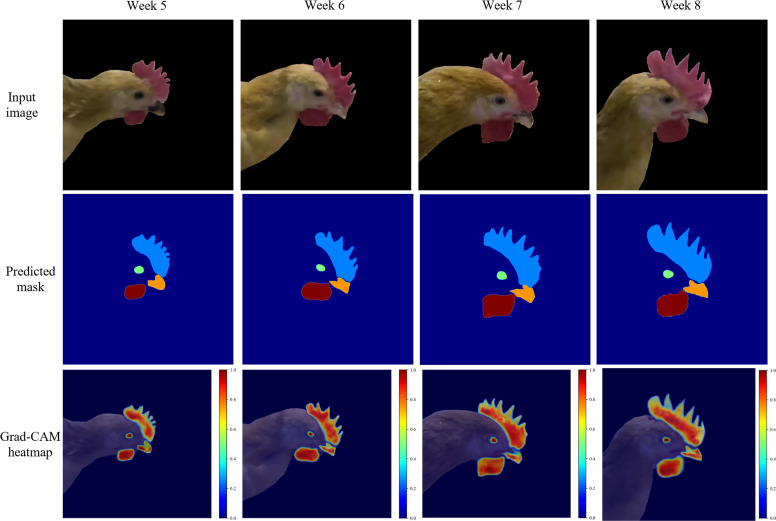
Visualization results of the CHSFormer model for chickens at different ages.

Fig. 7 shows the input RGB image, the class masks predicted by the model, and the heatmap generated by Grad-CAM visualization (Selvaraju et al., 2016), respectively. It can be observed that CHSFormer is able to accurately segment the comb, eye, beak, and wattle, with the predicted class masks closely matching the actual locations of the key chicken head regions. By comparing the input images and predicted masks of chickens at different ages, it is found that the boundary between the base of the beak and the head is not clear, is often covered by feathers, and may also be affected by the proximity of the comb to the edge of the beak. Nevertheless, the model successfully distinguishes the boundary between the beak and the comb during segmentation, with no cross-misclassification between regions, resulting in clear segmentation boundaries and distinct class separation. The Grad-CAM visualizations of chickens at different ages highlight the main attention areas of the model during inference, which indicates that the model can effectively focus on the discriminative regions relevant to the segmentation of key chicken head regions while suppressing background interference.

**Fig. 8:**
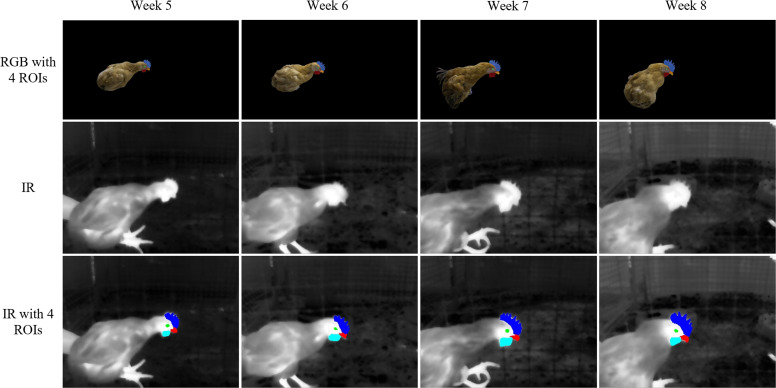
Results of mapping the key chicken head regions from the RGB image to the IR image based on CCMA.

**Fig. 9:**
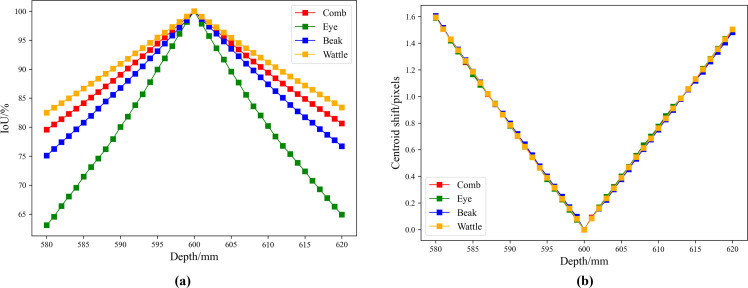
IoU and centroid offset of key chicken head region masks under different depth settings of the CCMA. (a) IoU of key chicken head region masks under different depth settings. (b) Centroid offset of key chicken head region masks under different depth settings.

***Results and analysis of CCMA*** Fig. 8 shows the results of mapping the key chicken head regions from the RGB image to the IR image based on CCMA. It can be observed that CCMA can accurately map the four key regions of chickens at different ages from the RGB images to the corresponding areas in the IR images. Additionally, Fig. 9 shows the impact of depth error on the accuracy of CCMA. As shown in Fig. 9(a), the greater the deviation of the depth value from 600 mm, the lower the IoU between the masks of each key region and those at the baseline depth, indicating that the overlap of the masks decreases as the depth error increases. Among the key regions, the IoU of the eye mask is the most sensitive to depth deviation. Fig. 9(b) shows that as the depth value deviates further from 600 mm, the centroid offset of the four key region masks also increases, with the trend being generally consistent across different regions. When the depth deviates by 20 mm from the baseline, the centroid offset can reach approximately 1.60 pixels. Overall, when the depth deviation is within 2 mm of the baseline, the IoU of all key chicken head region masks remains above 97 %, and the maximum centroid offset is only 0.17 pixels, which is much smaller than the size of a single pixel in the IR image. Given that the temperature distribution in the IR image is continuous and the temperature change between adjacent pixels is gradual, such minor mask shifts do not have a significant impact on the temperature statistics. Therefore, a depth error threshold of 2 mm was set in this study, which is sufficient to ensure the accuracy and scientific validity of subsequent temperature measurements.

### Analysis of heat stress indicator measurement results

**Fig. 10:**
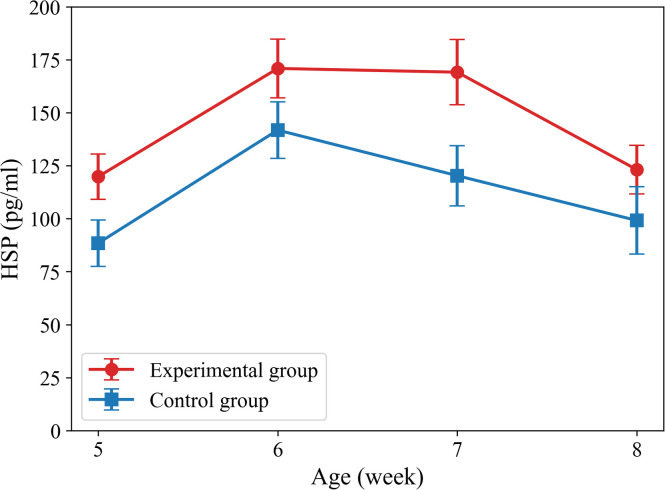
HSP levels in the serum of chickens of different ages in the experimental and control groups.

The concentrations of serum HSPs of chickens at different ages were measured, as shown in Fig. 10. The concentrations of serum HSPs in the experimental group were higher than those in the control group at all ages, which indicated that the high-temperature environment effectively induced the expression of heat shock proteins. The trends in the concentrations of serum HSPs for both the experimental and control groups were similar, with both reaching a peak at week 6. At week 7, the difference in the concentrations of serum HSPs between the experimental and control groups was the greatest. This suggests that changes in the concentrations of serum HSPs are influenced not only by heat stress but may also be related to the developmental stage of the chickens. Similar dynamic trends in HSPs have also been reported (Murugesan et al., 2017). Additionally, while this study did not further focus on the concentrations of serum HSPs under different rearing densities, prior research indicates that under heat stress conditions, higher rearing densities generally exacerbate stress responses, leading to elevated HSP levels due to reduced space for heat dissipation and increased competition for resources (Goo et al., 2019; Houshmand et al., 2012). The p-values for the t-tests of the concentrations of serum HSPs at different ages were all less than 0.001, which indicated highly significant statistical differences. These results fully demonstrate that the chickens in the experimental group developed a pronounced heat stress response under high-temperature conditions, providing a physiological basis for temperature measurement and heat stress assessment.

### Analysis of temperature measurement results in key chicken head regions

**Fig. 11:**
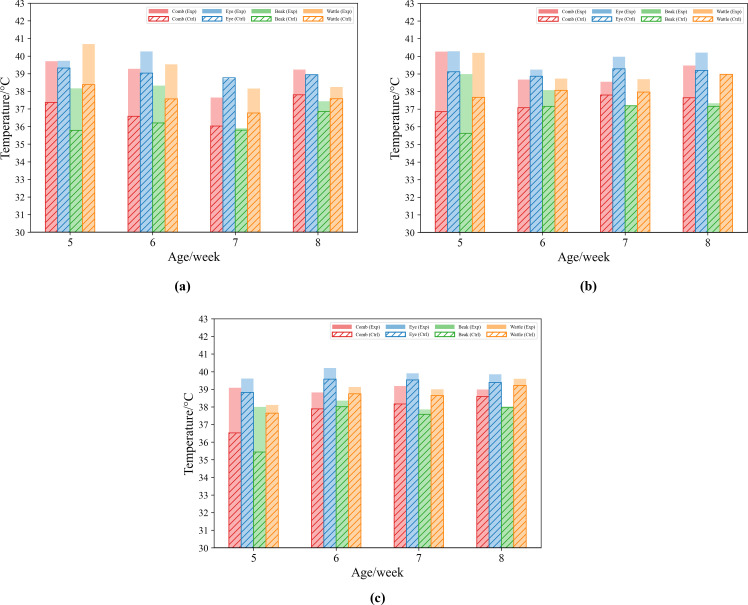
Comparison of key head region temperatures in experimental and control chickens of different ages under three rearing densities. (a) 4 bird/m^2^. (b) 8 bird/m^2^. (c) 12 bird/m^2^.

The mean temperatures of the comb, eye, beak, and wattle of chickens were plotted under different rearing densities and ages, as shown in Fig. 11. As shown in Fig. 11(a), under low rearing density, the temperatures of the comb, eye, beak, and wattle in the experimental group were mostly higher than those in the control group at different ages. For the same head region, the temperature in the experimental group was up to 2–3 °C higher than in the control group. The temperature differences in the comb and wattle between the experimental and control groups were significantly greater than those in the eye and beak. As shown in Fig. 11(b), under medium rearing density, the temperatures of the key regions in the experimental group were generally slightly higher than those under low rearing density, and the differences with the control group remained significant. The temperature difference in the comb between the experimental and control groups exceeded 1.0 °C from weeks 5 to 8, with the comb temperature in the experimental group peaking at week 5 and being more than 3 °C higher than that of the control group at the same age. This suggests that, under high-temperature conditions, chickens direct more heat to the featherless regions of the head through vasodilation (Yehia et al., 2025). As shown in Fig. 11(c), under high rearing density, the temperature trends of the key regions in the experimental and control groups were similar to those under medium and low densities. However, at higher ages, the temperature differences between the experimental and control groups for each key region decreased, which indicated that higher density increased the local microenvironment temperature of the chickens and reduced the efficiency of heat dissipation from the body surface. The physiological regulatory mechanisms for individual heat stress response were less able to offset the continuous accumulation of heat at the group level (Cengiz et al., 2015).

Overall, regardless of rearing density, the temperatures of the key head regions in the experimental group were mostly higher than those in the control group, which is consistent with previous research findings (Kim et al., 2021). Moreover, the temperature differences in the comb and wattle between the experimental and control groups were greater than those in the eye and beak. The results confirm that the temperatures of the comb and wattle increase significantly under high-temperature conditions and can effectively reflect the heat stress status of chickens.

## Conclusions

This study proposed the RGB-IR cross-modal collaborative segmentation framework for the accurate temperature measurement of key chicken head regions. The framework integrated three key components, which included the Mask2Former model for background segmentation, the CHSFormer model for key chicken head region segmentation, and the CCMA for cross-modal coordinate mapping. The Mask2Former achieved an MIoU of 99.05 % and an MPA of 99.47 %. It successfully segmented chickens from the background and provided high-quality, background-free RGB images for the key chicken head region segmentation task. The CHSFormer achieved an MIoU of 93.21 % and an MPA of 96.49 %. It accurately segmented the comb, eye, beak, and wattle in the RGB images and provided reliable ROIs for the implementation of CCMA. Furthermore, the CHSFormer significantly outperformed other mainstream segmentation models such as SegFormer and Deeplabv3 in both MIoU and MPA. The CCMA successfully mapped the segmented ROIs to the IR images with high spatial fidelity. This precise alignment established a reliable foundation for subsequent temperature measurement. The temperatures of the comb and wattle significantly increased under high-temperature environments compared to normal-temperature conditions. The temperature differences effectively reflected heat stress in chickens. Compared with previous methods such as box-based ROI selection and manual annotation, the approach proposed in this study shows significant advantages in both the accuracy and efficiency of extracting temperatures of the key chicken head regions. However, the approach requires manual positioning of chickens during image acquisition, which limits its scalability in dynamic farm environments. Future research could incorporate a depth camera to accurately measure the distance between the chicken and the RGB-IR camera, thereby enabling the framework to compensate for distance variations and adapt to temperature measurements of chickens at different distances in real-world farms.

## CRediT authorship contribution statement

**Yilei Hu:** Writing – original draft, Software, Methodology, Investigation. **Jinming Pan:** Resources, Conceptualization. **Lin Yu:** Visualization, Validation. **Di Cui:** Writing – review & editing, Supervision.

## Disclosures

The authors declare no conflicts of interest.

## Appendix Supplementary materials

Image, application 1
