# Supplementary material for: Heat stress assessment in chickens via key head region temperature measurement using semantic segmentation and cross-modal RGB-IR collaboration
Source: Poult Sci. 2025 Nov 24;105(1):106151. doi: 10.1016/j.psj.2025.106151 (PMC12720361; doi:10.1016/j.psj.2025.106151)
Supplement: Supplementary file 1 [file mmc1.docx]

**Table S1** Mean body weights of chickens across different age weeks

| Weeks of age/w | Body weights/g | |
| --- | --- | --- |
|  | Experimental Group | Control Group |
| 5 | 675.8 ± 12.5 | 684.3 ± 10.1 |
| 6 | 795.4 ± 12.4 | 787.9 ± 15.7 |
| 7 | 949.4 ± 8.3 | 958.2 ± 11.9 |
| 8 | 1163.8 ± 9.8 | 1178.9 ± 7.6 |
